# Patient engagement in the design of clinical research in Noonan syndrome spectrum disorders: a scoping review

**DOI:** 10.1186/s13023-021-02083-x

**Published:** 2021-10-26

**Authors:** Dagmar K. Tiemens, Jacqueline Nugteren, Erika Leenders, Ellen Wingbermühle, Carina A. C. M. Pittens, Jos M. Th. Draaisma

**Affiliations:** 1 Department of Pediatrics, Amalia Children’s Hospital, Radboud Institute for Health Sciences, Radboud University Medical Center, Nijmegen, The Netherlands; 2 Department of Human Genetics, Radboud University Medical Center, Nijmegen, The Netherlands; 3 Centre of Excellence for Neuropsychiatry, Vincent Van Gogh Institute for Psychiatry, Venray, The Netherlands; 4 Donders Institute for Brain, Cognition and Behavior, Radboud University Nijmegen, Nijmegen, The Netherlands; 5 Faculty of Science, Athena Institute, VU University, Amsterdam, The Netherlands; 6 Dutch Noonan Syndrome Foundation, Nijkerk, The Netherlands

**Keywords:** Noonan syndrome spectrum disorders, RAS/MAPK pathway, Patients, Participation, Level of engagement, Research, Ladder of Arnstein

## Abstract

**Background:**

Noonan syndrome spectrum disorders are a group of disorders caused by mutations in several genes of the RAS/MAPK pathway. Because of a highly heterogeneity and variable phenotypical manifestations of the disorders, these children and adults have a variable number of symptoms. Inclusion of their perceived experience of their health and developmental problems in research (design) could contribute to increased relevance of the research process and outcomes. The aim of this study is to get insight in what way patients with a Noonan syndrome spectrum disorder have been involved in the research process in order to learn for future engagement practices.

**Methods and results:**

To that end, the degree of engagement was measured by the eight levels of the participation ladder of Arnstein. Using a scoping review approach, 18 articles were selected in which patient engagement in the design of studies in patients with Noonan syndrome spectrum disorders was described over the past twenty years. Six of these articles reported engagement on the level of informing (level 3), 8 on the level of consultation (level 4), 2 on the level of placation (level 5)and 2 on the level of partnership (level 6).

**Conclusions:**

The current results do show a positive albeit still modest development of patient engagement over the last few years. A promising way to stimulate engagement is aiming to yield insights in the most important patients’ needs by developing a patient guided research agenda. However, this is not automatically followed by patient engagement at higher levels of participation in subsequent research steps. For this reason, in the Netherlands for example, a Dutch Noonan syndrome spectrum disorders research agenda is being developed, in a collaboration between the Dutch Noonan Syndrome Foundation and national scientific and clinical professionals.

## Introduction

Noonan syndrome spectrum disorders are a group of of phenotypically related conditions, resembling Noonan syndrome, characterized by a constitutional dysregulation of the Ras/mitogen-activated protein kinase (Ras/MAPK) signaling pathway and a highly variable expressivity [1, 2]. One out of 1.000 to 2.500 live births has a RASopathy syndrome [3, 4]. Classically, these syndromes have been described as clinically distinct syndromes, including Noonan syndrome (NS; MIM#163950), cardiofaciocutaneous syndrome (CFCS; MIM#115150), Costello syndrome (CS; MIM#218,040), Noonan syndrome with multiple lentigines (NSML; MIM#151100) and Noonan-like syndrome with loose anagen hair (NS-LAH; MIM#607721), with clinical overlap between the different syndromes [1]. At this moment, at least 19 genes have been found to be associated with the Noonan syndrome spectrum disorders [1].

During the last decades, the perspectives of patients, caregivers and disease advocacy organisations have become increasingly important in the design, planning and execution of health research [5, 6]. Experiential knowledge of patients can be complementary to the expert knowledge of researchers, or can give a different perspective on current knowledge (*substantive arguments*) [5, 7]. Additionally, patients can be considered ‘end users’ of developed knowledge and should therefore be involved in medical scientific research affecting them (normative argument) [8]. Finally, prioritizing their problems and complaints gives patients a say in what is felt the most important. Such an active role for patients, care givers and disease advocacy organisations in the development and execution of research can increase the amount of research on that specific condition and enhance the support for and participation in studies (*political arguments*) [9].

Integrating patients’ experiences in health research policy making—particularly in a research agenda setting—could provide for a wider perspective to health research and care, and thereby contribute to increased quality and relevance of studies [5, 6, 10]. Especially for patients with a Noonan syndrome spectrum disorder the inclusion of patients’ experiences is relevant since the heterogeneity and variable phenotypical manifestations of these disorders leads to a variable number of symptoms and complaints. Alignment of the experiences and needs of patients to research results in more relevant research process and outcomes.

In 2014 a systematic review was published on approaches for engaging patients in clinical research on rare diseases [11]. In this review no publications were reported of the engagement of patients, caregivers or patient organizations into the planning and/or conduct of research on one of the different Noonan syndrome spectrum disorders.

This article aims to explore how and to what degree individuals with a Noonan syndrome spectrum disorder (patients), their relatives and their representatives, were engaged in clinical research since the first gene associated with Noonan syndrome was detected in January 2001. To provide for an overview of engagement, various aspects of research were examined: type of clinical research, various ways of patient representation and the six steps of the research process, using the model of Rummel and Ballaine [12]. The participation Ladder of Arnstein, an eight level tool to measure degrees of engagement and decision making power, was used to clarify the way patients were engagement in each of these aspects [13]. The information this provides may be helpful to improve patient engagement in future research and thereby contribute to increased relevance of research outcomes to patients with a Noonan syndrome spectrum disorder.

## Materials and methods

### General

This study adopted a scoping review approach. The scoping review (or scoping study) is a strategy designed to map literature in a research area, identifying key concepts, sources of evidence, and research gaps [14, 15]. Scoping reviews are particularly helpful in studying literature in research areas with emerging evidence. The methodology employed in this study was drawn from the commonly-used framework proposed by Arksey and O’Malley and advanced by Levac, Colquhoun and O’Brien [14, 15]. This rigorous approach involves five key stages: (stage 1) identifying the research question, (stage 2) identifying relevant studies, (stage 3) study selection (this study used the PRISMA Flow chart), (stage 4) data extraction, and (stage 5) collecting, summarizing and reporting the results.

Search results were limited to English, Dutch and German texts and publication date from January first 2001 through July 2020, because of the discovery of the first Noonan syndrome related gene in January 2001 [16]. Search results were limited to (development of) clinical studies. Articles about proceedings of a symposium, in which patients or their representatives and the development and implementation of a management guideline were included in this search as well, as clinical guidelines can be considered the result of scientific research, the discussion based on it and subsequent opinion formation [17]. Grey literature was not included.

### Identifying the research questions

The research questions addressed in this scoping review were defined as:

1. In what kind of clinical research have patients, their relatives and/or their representatives been engaged? (Article characteristics, type of article and year of publication).
2. In what way have patients been represented?
3. What was the level of engagement in each of the six research steps as defined by Rummel and Ballaine [12]?
4. What was the level of engagement according to the ladder of Arnstein [13] and did the degree or level of engagement differ looking at type of clinical research and at each of the six research steps?
5. Did the level of engagement change over time?

### Identifying relevant studies

Five electronic databases (PubMed, Embase, Web of Science, Medline and Google scholar) were searched using the following Boolean search strategy identified through input from the research team and consultation of the university-affiliated librarian: (type of) Noonan syndrome spectrum disorder AND (means of) engagement AND patient and their synonyms. Both subject headings (such as MeSH) and free text terms were applied. The initial search was on 17 July, 2020. The search strategy for Pubmed is provided in Table 1.

**Table 1:** The search strategy for Pubmed

Noonan syndrome spectrum disorder	(Type of) engagement	Patient and their representation
Noonan syndrome Noonan Costello syndrome Cardiofasciocutaneous syndrome Cardio-facio-cutaneous syndrome CFCS Loose Anagen Hair syndrome Loose anagen hair Leopard syndrome LEOPARD syndrome RASopathy	Community participation Policy making Decision making Patient participation Cooperative behaviour Cooperative Recruit Volunteer Empowerment Participation Participate Shared decision Patient activation Engagement Collaboration Cooperation	Patient Patients Family Parent Parents Relatives Patient federation Patient representative organisation

### Study selection

Two authors independently screened all article titles and abstracts to determine eligibility for full text review addressing the research questions. Discrepancies between reviewers were resolved by discussion and consensus or involvement of a third reviewer. Full texts of all remaining studies were retrieved and assessed independently by the same authors based on the same criteria and methods applied in title and abstract screening. To supplement this search, reference lists of reviews and included studies were scanned (so-called snow-balling strategy), and the “related articles” feature of Pubmed was used. Search results were collected and deduplicated in Endnote and then exported into Rayyan software for ease of management [18]. No methodological quality assessment was performed, as scoping reviews aim to map existing evidence and not to present a judgement regarding the ‘weight’ of evidence [14].

### Data extraction

One author analysed the data of all included studies. The data abstraction chart was drafted collectively and validated independently by one other reviewer in a random sample of 10 articles, after which additional categories were added in an iterative process. The final form included the following criteria:

#### Article characteristics

Authors and year of publication.

#### Types of articles

These were distinguished according to its content in an iterative process.

1. Research: Articles describing the results of a clinical study
2. Proceedings: Articles describing proceedings of a meeting/congress
3. Guidelines: Articles describing clinical management guidelines

#### Ways in which patients were represented

These were defined as:

1. Individuals with a Noonan syndrome spectrum disorder (patients)
2. Patients represented by relatives
3. Patient representative organizations (including family support groups and disease advocacy organisations)

#### Research step 1 to 6

These steps were defined using the model of Rummel and Ballaine [12].

1. Step 1: identifying the general study
2. Step 2: choosing the research topic
3. Step 3: formulating plan and technology
4. Step 4: collecting data
5. Step 5: analyzing and interpreting data
6. Step 6: writing content and other ways of presenting the findings

In every step patients or patient representative groups may be involved. Involvement of recruitment of patients was interpreted as part of step 4. Involvement in development and publication of a guideline may be seen as step 5 and 6.

#### Levels of engagement

**Fig. 1:**
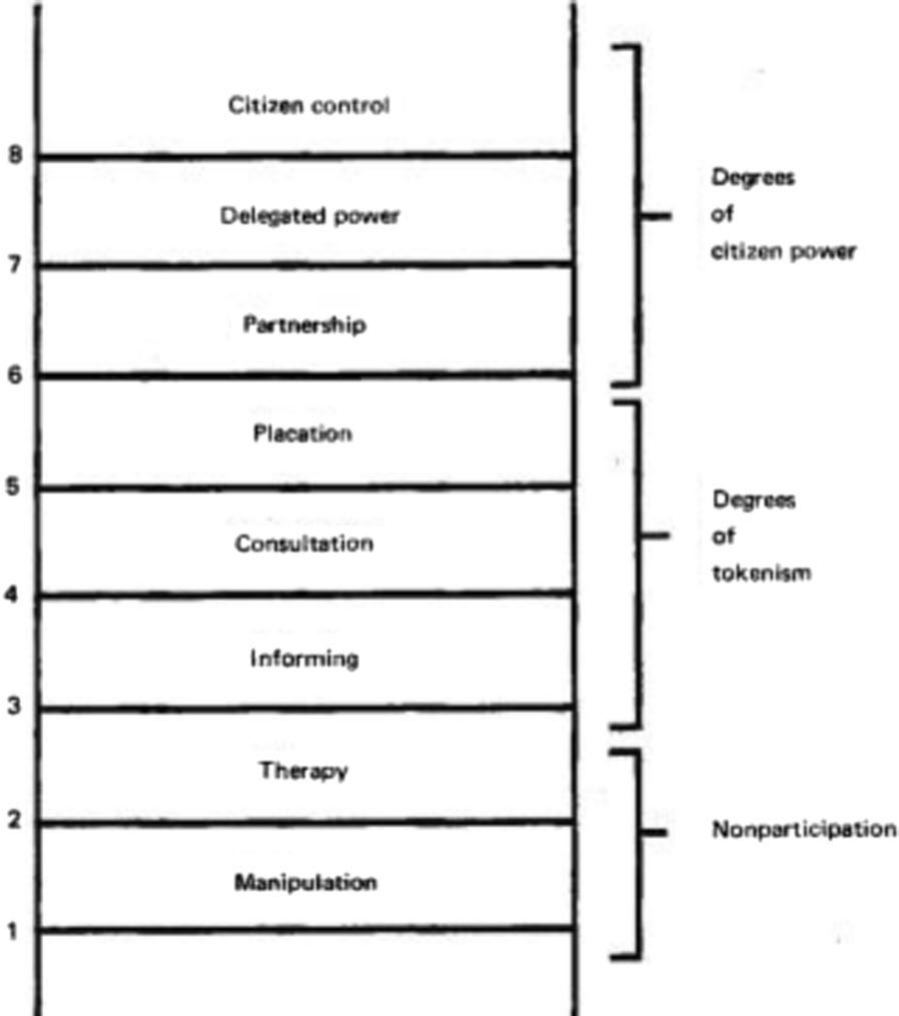
Arnstein’s ladder of participation

To measure the level or degree of engagement of the patients and representatives in the included articles, Arnstein’s ladder of citizen participation was used (Fig. 1) [13]. This approach distinguishes between so-called “powerholders” on one hand with unlimited access to information and decision making power (the researchers in the analyzed studies) and the so-called “powerless” with limited access to information and decision making power (patients and/or their representatives).

The ladder distinguishes 8 levels of participation and decision making power of the “powerless”. The bottom levels of the ladder are (1) manipulation or education and (2) therapy or curing. In this context, these levels describe the participation of patients as objects of study, and not as being actively engaged in the development and execution of the research process. Studies with engagement at these levels were excluded. Levels (3), (4) and (5) progress to levels of “tokenism” that allow the participants to be “informed”, but without a channel of feedback (level 3) or to have a voice in consultation (level 4). In studies with participation at these levels, patients may indeed hear and be heard, but they lack the power to ensure that their views will be used by the powerholders. For example, in this context, level 3 includes the engagement of disease advocacy groups in patient recruitment.

Further up the ladder are levels with increasing degrees of decision-making power. At level 5 (placation), patients may advise or plan ad infinitum, but researchers (“traditional powerholders”) retain the right to judge the legitimacy or feasibility of the advice. Patients can enter into a partnership (6) that enables them to negotiate and engage in trade-offs with the researchers. At the topmost steps, (7) delegated power and (8) citizen control, patients obtain the majority of decision-making seats, or full managerial power [8].

Finally, to examine the level of engagement within the categories, the level on the ladder of Arnstein per subtype within each category was determined.

## Results

### Identifying relevant studies and study selection

After searching 5 databases (Pubmed, Cochrane, Embase, Web of science and Google scholar), 383 articles met previously described search terms. These articles were deduplicated, which led to 300 individual articles. The article titles and abstracts of these articles were uploaded in Rayyan and screened.

**Fig. 2:**
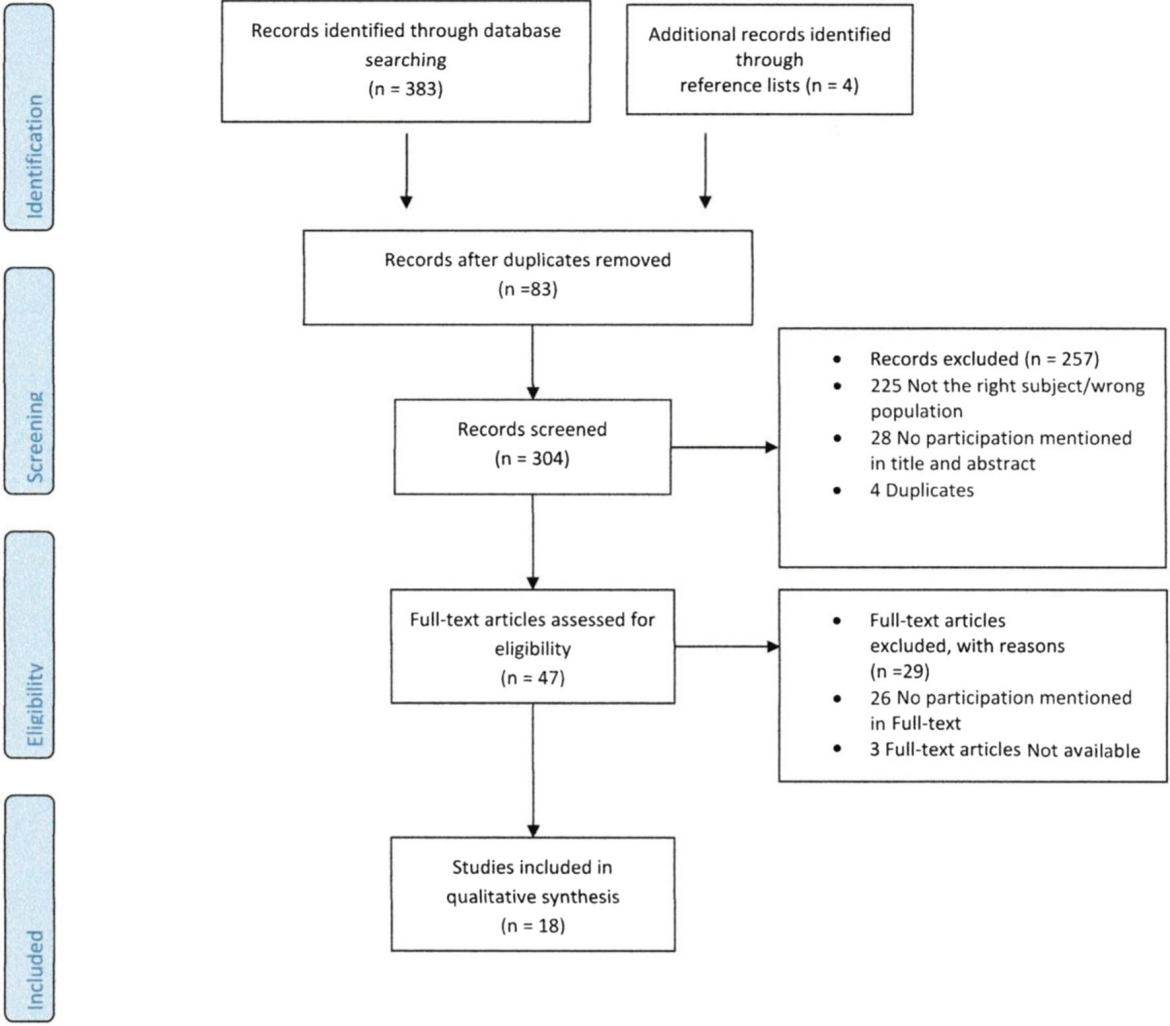
Prima flowchart for study selection

They were first screened on relevance and inclusion criteria according to title and abstract, which excluded 257 articles and then on full article, which excluded 29 articles. The 14 articles that were left, were included in this review. Four additional articles could be included after scanning of the reference lists of the included articles (Fig. 2), which led to 18 included articles in total.

The characteristics of the included articles are presented in Table 2, and further elaborated on the next sections.

**Table 2:** Characteristics of included articles in chronological order

Author(s) [ref]	Year of publication	Type and title of article	Type of patient representation	Research step	Highest degree of engagement (Arnstein)
Axelrad [19]	2004	Clinical study CS Adaptive skills, cognitive and behavioral characteristics of Costello syndrome	Patient representative organisation	Collecting data	Informing
Gripp [23]	2007	Clinical study CS and CFC Further delineation of the phenotype resulting from BRAF or MEK1 germline mutations helps differentiate cardio-facio-cutaneous syndrome from Costello syndrome	Patient representative organisation	Collecting data	Informing
Rauen [30]	2008	Proceedings CS Research Symposium Molecular aspects, clinical aspects and possible treatment modalities for Costello syndrome: Proceedings from the 1st International Costello Syndrome Research Symposium	Patients Relatives representing patients Patient representative organisation	Choosing research topic Writing content and other ways of representing findings	Consultation
Rauen [31 ]	2010	Proceedings symposium on the Ras/MAPK pathway Proceedings from the 2009 genetic syndromes of the Ras/MAPK pathway: From bedside to bench and back	Patients Relatives representing patients Patient representative organisation	Choosing research topic Writing content and other ways of representing findings	Consultation
Romano [3]	2010	Guidelines as a result of a conference NS Noonan syndrome: clinical features, diagnosis, and management guidelines	Patient representative organisation	Analyzing and interpreting data Writing content and other ways of representing findings	Partnership
Pierpont [ 29]	2014	Guidelines as a result of a conference CFCS Cardio-facio-cutaneous syndrome: clinical features, diagnosis, and management guidelines	Patient representative organisation	Analyzing and interpreting data Wwriting content and other ways of representing findings	Partnership
Rauen [4]	2015	Proceedings symposium NF and RASopathies Recent developments in neurofibromatoses and RASopathies: management, diagnosis and current and future therapeutic avenues	Patients Relatives representing patients Patient representative organisation	Choosing research topic Writing content and other ways of representing findings	Consultation
Korf [27]	2015	Proceedings of International Meeting on Rasopthies The third international meeting on genetic disorders in the RAS/MAPK pathway: towards a therapeutic approach	Patients Relatives representing patients Patient representative organisation	Choosing research topics Formulating plan and technology Writing content and other ways of representing findings	Consultation
Niemczyk [28 ]	2015	Clinical study NS Incontinence in persons with Noonan Syndrome	Patient representative organisation	Collecting data	Informing
Johnson [ 26]	2015	Clinical study CF and CFC Function and disability in children with Costello syndrome and Cardiofaciocutaneous syndrome	Patient representative organisation	Collecting data	Informing
Croonen [20 ]	2016	Clinical study NS Perceived motor problems in daily life: Focus group interviews with people with Noonan syndrome and their relatives	Patient representative organisation	Collecting data Aanalyzing and interpreting data	Consultation
Templin [34]	2016	Clinical study CFC Prenatal findings in cardio-facio-cutaneous syndrome	Patient representative organisation	Collecting data	Informing
Stevenson (33]	2016	Proceedings of International Symposium on Rasopthies Function and disability in children with Costello syndrome and Cardiofaciocutaneous syndrome	Relatives representing-patients Patient representative organisation	Choosing research topics Writing content and other ways of representing findings	Consultation
Garg [21]	2017	Clinical study NS, CS and CFC Autism spectrum disorder and other neurobehavioural comorbidities in rare disorders of the Ras/MAPK pathway	Patient representative organisation	Collecting data	Informing
Grant [22]	2018	Clinical study NS Reclassification of the BRAF p.Ile208Val variant by case-level data sharing	Relatives representing patients	Choosing research topic	Consultation
Rauen [32]	2018	Proceedings International RASopathies symposium Proceedings of the fifth international RASopathies symposium: When development and cancer intersect	Patients Relatives representing patients Patient representative organisation	Choosing research topic Writing content and other ways of representing findings	Consultation
Gross [25]	2020	Proceedings of meeting to discuss advancing RAS/ RASopathv therapies Advancing RAS/RASopathy therapies: An NCI-sponsored intramural and extramural collaboration for the study of RASopathies	Patients Relatives representing patients Patient representative organisation	Choosing research topics Formulating plan and technology Writing content and other ways of representing findings	Placation
Gripp [24]	2020	Proceedings of international meeting on RASopathies The sixth international RASopathies symposium: Precision medicine-From promise to practice	Patient representative organisation	Choosing research topic Writing content and other ways of representing findings	Placation

Some of the characteristic examples of the level of engagement of the different articles are given in Table 3.

**Table 3:** Some of the characteristic example of level of engagement as cited in the different articles

Author(s) [ref]	Year of publication	Characteristic example of level of engagement
Axelrad [19]	2004	“Through the 3rd International Costello Syndrome Conference, families attending the conference whose child had a clear diagnosis of Costello Syndrome and was between age 2 and 21 years were contacted for participation in this research study”
Gripp [23]	2007	“Patients clinically diagnosed with Costello syndrome were identified at the 2003 and 2005 International Costello Syndrome Meetings, through the Costello Syndrome Family Network and through physician referral”
Rauen [30]	2008	“Patients clinically diagnosed with Costello syndrome were identified at the 2003 and 2005 International Costello Syndrome Meetings, through the Costello Syndrome Family Network and through physician referral”
Rauen [31 ]	2010	“This unprecedented NIH-sponsored symposium was held in conjunction with family conferences sponsored by the Noonan Syndrome Support Group (NSSG), the Costello Syndrome Family Network (CSFN) and the International Costello Syndrome Support Group (ICSSG), CFC International”
Romano [3]	2010	“ The Noonan Syndrome Support Group convened a conference of health care providers, all involved in various aspects of NS, to develop these guidelines for use by pediatricians in the diagnosis and management of individuals with NS and to provide updated genetic findings”
Pierpont [ 29]	2014	“To address this need, CFC International, a nonprofit family support organization that provides a forum for information, support, and facilitation of research in basic medical and social issues affecting individuals with CFC, organized a consensus conference”
Rauen [4]	2015	“The international symposium “Recent Developments in Neurofibromatoses and RASopathies: Management, Diagnosis and Current and Future Therapeutic Avenues” was attended by clinicians, basic scientists, physician-scientists, clinical and molecular geneticists, advocate leaders, genetic counselors, trainees, students and individuals with Ras/MAPK syndromes and their families”
Korf [27]	2015	“Parent and patient advocates opened the meeting with a panel discussion to set the stage regarding their hopes and expectations for therapeutic advances”
Niemczyk [28 ]	2015	“Nineteen children (5–17 years) and 10 adults (18–48 years) with NS were recruited through a German parent support group”
Johnson [ 26]	2015	“Participants were recruited from the International Costello Syndrome Family Forum and the CFC International Conference in Orlando, Florida in August 2013. Participants with Noonan syndrome were also recruited at the conference”
Croonen [20 ]	2016	“The study was conducted in collaboration with the Dutch Noonan syndrome patient association. Participants were recruited through an announcement for one of three planned focus group interviews on the flyer of the Noonan syndrome contact day” “The analyzed data were presented on the NS contact day. All attendees agreed with the results without any additional remark”
Templin [34]	2016	“Parents of CFC patients were contacted through referring clinicians and the family support group”
Stevenson (33]	2016	“The symposium is unique in its approach as it is held in conjunction with multiple advocacy meetings for RASopathy family/patient support groups. A poster session of submitted scientific abstracts and invited posters for representative parent/patient support groups was held to open the symposium allowing for interaction and discussion between the lay community and the scientific community”
Garg [21]	2017	“The study was also advertised on newsletters, family information days, and on the social media of Noonan, Costello, and CFC syndrome charities and the Noonan Syndrome Association (UK) and Costello Kids for their help with recruitment”
Grant [22]	2018	“Our laboratory was contacted by the mother of Proband 1”
Rauen [32]	2018	“Clinicians, basic scientists, physician-scientists, advocate leaders, trainees, students, and individuals with RASopathies and their families attended” ..The patient and family advocates strongly requested the researchers and medical experts to stay interested in issues that adults live with and to continue to heed advocacy support groups”
Gross [25]	2020	“A multidisciplinary group of 38 care providers, basic scientists, and representatives from the patient advocacy group RASopathiesNet……… aimed to define the RASopathies and RASopathy‐associated genes for the purposes of ART, identify the available tools for translational research in the RASopathies, describe the longitudinal cohort study that is the cornerstone of ART, and discuss potential solutions for the various challenges that have hindered investigators from initiating therapeutic clinical trials for individuals with RASopathies to date Outreach activities through RASopathiesNet and the syndrome‐specific family groups have indicated that individuals with a RASopathy are eager to engage in the scientific process”
Gripp [24]	2020	“This meeting brought together basic science researchers, clinicians, clinician scientists, patient advocates, and representatives from pharmaceutical companies and the National Institutes of Health. Novel RASopathy genes, variants, and animal models were discussed in the context of medication trials and drug development. Attempts to define and measure meaningful endpoints for treatment trials were discussed, as was drug availability to patients after trial completion”

### In what kind of research have patients and/or their representatives been engaged?

Out of the 18 selected articles, eight (47%) described the results of a clinical research study that was performed, eight articles described the proceedings of a symposium/meeting and two articles described the development of guidelines. From the eight articles with a clinical research study, three articles [19, 21, 26] presented results about (neuro)behavioral aspects, two about motor problems [20, 26], one about incontinence [28], one about prenatal findings [34], one about further delineation of a phenotype [23] and one about reclassification of a gene variant [22]. There were eight articles in which the proceedings from a RASopathy symposium/meeting were described [4, 24, 25, 27, 30–33], of which one article described the proceedings from of a Costello syndrome research symposium [30]. Two articles described guidelines as a result on a symposium on NS and CFCS [3, 31].

### In what way have patients been represented in the included articles?

In seven of the 18 articles in which the results of a research study were described, recruitment took place through a patient representative organization [19–21, 23, 26, 28, 34]. For example, patients were recruited at the 2003 and 2005 International Costello syndrome meetings [19, 23]. For a study on incontinence in patients with Noonan syndrome in Germany, 19 children (5–17 years) and 10 adults (18–48 years) were recruited through a German parent support group [28]. In the eighth article, the authors were contacted by a mother with a specific (research) question about the pathogenicity of a *BRAF* variant [22].

In the eight articles in which the proceedings of a meeting/symposium are described, patients and/or patient representative organizations participated explicitly. The first time this was reported concerned the 1st International Costello Syndrome Research Symposium in 2007 [30]. This symposium brought clinicians, scientists and individuals with Costello syndrome and their families together.

Two articles described (the development of) a guideline, which was developed together with a patient representative organization [3, 31]. Although there was no patient involvement in the design of Noonan syndrome spectrum diseases clinical studies underlying the guidelines, the patient representative group was a partner in the interpretation of study results and the implementation of these results in the guidelines.

### How was the engagement in each of the six research steps?

We found no article that included patient engagement in the entire research trajectory. Eight of the articles reported about engagement of patients/patient representatives organizations in the choice of the research topic (research step 2) [4, 24, 25, 27, 30–33]. In two articles patients were involved in the formulation of the plan and the technology of the clinical research study (research step 3) [25, 27]. In one of these studies the results of an initiating meeting were presented [25]. The participants aimed to define the non-NF1 RASopathies and RASopathy‐associated genes for the purposes of Advancing RAS/RASopathy Therapies, identify the available tools for translational research in the non-NF1 RASopathies, describe the longitudinal cohort study, and discuss potential solutions for the various challenges that have hindered investigators from initiating therapeutic clinical trials for individuals with non- NF1 RASopathies to date.

Seven out of the 18 articles (39%) reported only participation in step 4 (recruitment of patients or data collection). For example, Axelrad et al. (2004) reported that patients for their study on adaptive skills, cognitive, and behavioral characteristics of Costello Syndrome were recruited by means of the 3rd International Costello Syndrome Conference [19].

Three articles reported participation in step 5, analyzing and interpretation of the data [3, 20, 31]. One study in addition to recruitment of patients [20]. They presented their study data on the Noonan syndrome contact day one year after the study was performed for interpretation and discussion on the data. Patient representative organizations were engaged in the process of analyzing and interpreting the data (research step 5) and writing the guidelines (research step 6) [3, 31]. In the eight articles in which the proceedings of a meeting/symposium are described, patients and/or patient representative organizations participated explicitly [4, 23, 25, 27, 30–33]. All these eight articles were also written by members of the patient representative organizations (research step 6).

### What was the level of engagement according to the ladder of Arnstein?

Six of the 18 articles reported participation on level 3 (informing) (33%), 8 on level 4 (consultation) (44%), 3 on level 5 (placation) (11%), and 2 on level 6 (partnership) (11%). There were no reports found on the two highest levels of participation (7 and 8).

In six out of eight of the articles about clinical research studies, participation took place at the level of informing (level 3). For example, in the article of Templin et al. (2016), prenatal features were extracted from a national database and additional data were collected from 16 families contacted and recruited through the French association of CFC-Costello syndrome [34]. In none of these six articles another engagement of the patient representative organization was mentioned than the help of the recruitment of patients to make a research study possible.

In two of the eight articles about clinical research studies, the (highest) level of participation was consultation [22, 26]. The study by Croonen et al. (2016) was conducted in collaboration with the Dutch Noonan Syndrome Foundation [26]. Participants were recruited through an announcement for one of three planned focus group interviews on the flyer of the Noonan syndrome contact day in 2013, where focus group interviews were conducted (information: level 3). There-after the analyzed data were presented to discuss the results on the Noonan syndrome contact day in 2014. As patients had the opportunity to hear the results and had the choice to make comments, engagement in this study was scored at level 4 (consultation). In another study the mother of a patient came to the laboratory with a request for a study, and was heard [22]. She received a report of a variant in *BRAF* following prenatal RASopathy testing. The variant had been previously classified by this laboratory as a Variant of Unknown Significance, which prompted reevaluation of the variant. Multiple sources of case-level data as well as the presence of the variant in the general population yielded sufficient evidence to reclassify the variant as likely benign. This reclassification alleviated significant concern for the family, and the child was born healthy with no clinical manifestations of Noonan syndrome or a RASopathy.

In the articles about the proceedings of symposiums the general level (six out of eight) of engagement was consultation (level 4). The first symposium/meeting reported on was the 1st International Costello Syndrome Research Symposium in 2007 [30]. The symposium occurred in conjunction with the Costello Syndrome Family Network (CSFN) conference bringing together clinicians, scientists, physician‐scientists, advocate leaders, trainees, students and individuals with Costello syndrome and their families. The overall goal of the symposium was to provide an open forum for researchers, clinicians, and physician‐scientists to share and discuss basic science and clinical issues setting forth a solid framework for future research, translational applications directed towards therapy and best practices for individuals with CS. At this symposium one of the patients presented the problems she encountered. This article about proceedings of a symposium was followed by more articles about the same type of symposia/meetings [4, 27, 31–33].

In two articles a higher level of participation, placation (level 5), could be found. In the article of Gross et al. (2020), the authors (including three members of the patient representative organization) describe the goals of the meeting [25]. However, the method of the participation of patients and their representatives in the follow-up process is not made clear [25]. In the article of Gripp et al. (2020), the authors (including eight members of different patient representatives organizations) emphasized the added values of the patient perspectives:”Patient advocates highlighted pain, plexiform neurofibromas, intractable seizures, neurocognitive function, and social skills as the most urgent treatment targets. Further studies on how RASopathies impact the lives of adults were requested [24].”

In two articles (including authors from the patient representatives organizations) guidelines for NS and CFCS were formulated [3, 31]. Although not a report of an original research study, these guidelines were formulated in a consensus meeting, coordinated by the patient representatives organizations with health care providers with expertise in various aspects of the disorders, with the aim of developing guidelines for diagnosis and clinical management. These reports are the result of those efforts and are intended to provide pediatricians and other specialists with information of key clinical features of NS and CFCS, to provide an update of currently understood genetic causes, and to present management recommendations. This may be seen as a form of partnership (level 6).

Although the number of articles of clinical studies with patient engagement is equal to the number of proceedings, the median score of engagement measured by Arnsteins’ participation ladder, as shown in Table 4, is the lowest in articles of clinical studies (informing). The highest level was found in articles that describe the development of guidelines (partnership). Articles describing proceedings had most often the level of consultation.

**Table 4:** Level of engagement per type of article, measured with Arnsteins’ participation ladder

Type of article	Number of studies	Scores on Arnsteins’ ladder
Clinical study	8	Informing (level 3): 5 times Consultation (level 4): 3 times
Proceedings	8	Consultation (level 4): 6 times Placation (level 5): 2 times
Guideline	2	Partnership (level 6): 2 times

Looking at the amount of the included clinical articles, patient engagement was seen most often in step 6 (writing content and other ways of presenting the findings) of the research process, followed by step 2 (choosing the research topic) and step 4 (collecting data). Only a few studies were found with patient engagement in step 5 (analyzing and interpreting data) and step 3 (formulating plan and technology).

However, looking at the level of engagement measured by Arnsteins’ participation ladder, the highest median level was found when patients were involved in analyzing and interpreting data (research step 5), with placation (level 5) (Table 5). Choosing the research topic (step 2) and writing content and other ways of presenting data (step 6) had a median level between consultation and placation (between level 4 and 5). Research step 4 (collecting data) showed the lowest median level of patient engagement (informing, level 3).

**Table 5:** Level of engagement in the six research steps in the included 18 articles, measured with Arnsteins’ participation ladder

Research step	Total of 18 articles	Level on Arnsteins’ ladder
Step 1 Identifying the general study	0	
Step 2 Choosing the research topic	9	Consultation (level 4): 7 times Placation (level 5): 2 times
Step 3 Formulating plan and technology	2	Consultation (level 4): 1 time Placation (level 5): 1 time
Step 4 Collecting data	7	Informing (level 3): 6 times Consultation (level 4): 1 time
Step 5 Analyzing and interpreting data	3	Consultation (level 4): 1 time Partnership (level 6): 2 times
Step 6 Writing content and other ways of presenting the findings	10	Consultation (level 4): 6 times Placation (level 5): 2 times Partnership (level 6): 2 times

### What was the change over time?

Over the last twenty years there is a modest, but clear increase in the amount of articles in which Noonan syndrome spectrum disorder patients participated in the process on research focusing on their health. From one study in 2000–2005, four studies in 2006–2010, five studies in 2011–2015 to eight studies in 2016–2020. Although the score of participation level (consultation, level 4) remained the same over the years (data not shown), the amount and diversity of the research steps patients were engaged has increased. (Table 6).

**Table 6:** Number of studies in which Noonan syndrome spectrum disorder patients were engaged in a particular step in research over the last twenty years

Year of publication	Total number of studies (total n)	Step 1 Identifying the general study (n)	Step 2 Choosing the research topic (agenda setting) (n)	Step 3 Formulating plan and technology (n)	Step 4 Collecting data (n)	Step 5 Analyzing and interpreting data (n)	Step 6 Writing content and other ways of presenting data (n)
2000–2005	1				1		
2006–2010	4		2		1	1	3
2011–2015	5		2	1	2		4
2016–2020	8		5	1	3	1	4

## Discussion

The aim of this scoping review was to get insight into what extent and in what way patients with a Noonan syndrome spectrum disorder and/or their representatives have been involved in one or more phases of clinical research and indicate the level of patient engagement in health research on Noonan syndrome spectrum disorders. This way, we hope to gain insight into how patients can become engagement more and better in future research and thereby having more impact on the process and outcomes of research. Although Forsythe et al. (2014) could not identify any articles with patient engagement in clinical research on Noonan syndrome spectrum disorders in 2014, we could identify five articles dated before 2014 with patient engagement in research, probably due to the use of another search strategy and/or other inclusion criteria [11]. Our scoping review could identify 18 articles with patient engagement in clinical research on Noonan syndrome spectrum disorders over the last 20 years.

The lowest median level of participation was found in clinical studies, even though the amount of clinical studies in the included articles was relatively high. No evidence was found that patients had participated in all six steps of the research process, though in recent years the number of articles which reports patient engagement have been increased, not only in quantity, but also in the variety and number of research steps. Only three studies were found with patient engagement in analyzing and interpreting data (research step 5), but when patients did engage in this step, the engagement level had the highest level (level 5, placation). The lowest level of engagement was found when patients were engaged in collecting data (step 4).

These findings suggest that patient engagement over the last two decades has mostly been at the level of informing (level 3 on the 8 levels of participation at the ladder of Arnstein) or consultation (level 4), by bringing the researchers and the patients and/or their representatives together. A positive finding is the observation that in recent years reporting about patient engagement seems to have been increased.

A social/cultural change of the position of patients may be a reason for the increase of engagement in the number of steps of the research process, the relatively high degree of patient empowerment when engagement takes place during the analyzing and data interpreting stage of the research process (step 5) or engagement in the development of management guidelines. This is also reflected in changes in policies and the emergence of institutes. For instance, in 2010 the Patient-Centered Outcomes Research Institute, an independent nonprofit, nongovernmental organization authorized by the congress was created in the United States. This institute funds studies that address methods for improving engagement, for evaluating the impact of engagement on research outcomes, and for assuring that study questions and outcomes are meaningful to patients [35]. Moreover, the European Reference Network Ithaca, in which the Noonan syndrome spectrum disorders are incorporated, is a patient centered network which meets the needs of those with rare congenital malformation and syndromes with intellectual and other neurodevelopmental disorders [36]. All activities of Ithaca include patients, their families and lay organizations as equal partners in a network aiming at developing best practices and initiating guideline development.

There are many patient representative organizations, for example the French Costello Group, CFC International, the Dutch Noonan syndrome Foundation, RASopathies Network and the Costello Syndrome Family Network, working together with clinicians and researchers. Patients, patient organizations, clinicians and researchers can work together in a more effective way to their mutual benefit. Not only with the goal of patient recruitment as is often done, but also to share and discuss basic science and clinical issues, setting forth a solid framework for future research, translational applications directed towards therapy and best practices for individuals.

More recently, patient representative organizations have become increasingly involved in the choice of the research topic and the formulation of a plan and methodology. Hopefully, the initiatives as formulated in the recent articles about the proceedings published by Gross et al. and Gripp et al. can lead to improved patient engagement in the whole research trajectory and with higher levels of participation according to the participation ladder of Arnstein, aiming at least for partnership (level 6) [24, 25].

## Limitations

The framework by Rummel and Ballaine was not used earlier in healthcare [12]. However, because of the simple and clear definitions of this process model we have decided to use this framework. Arnstein’s ladder is a well-known model displaying different levels of participation, ranging from manipulation to citizen control. Abma and Broerse stated that the ladder easily translates to the health field, although it only displays levels of participation without specifying how they may be achieved [37]. However, this was not the aim of our study.

The assessment of articles relied heavily on the content of the titles and abstract which were used for initial inclusion in this study. Often, the impact of engagement was not worked out, but only the engagement in the process was described. Moreover, the interpretation of the level of engagement was sometimes not as clear as described in this article, e.g. one can discuss if the patient recruitment via the patient organization is the same as collecting data in the research steps, and can be seen as a real engagement. Also, an higher level of engagement, according to the ladder of Arnstein, does not automatically mean that patients and / or their representatives are better engaged in the process.

## Future implications

The current results do show a positive albeit still modest development of patient engagement over the last few years. However, it is important to continue and expand this development. Engagement in clinical research seem to be relatively high given the numbers, but is relatively low looking at the level of participation, with a score of level 3, informing. In contrast, the engagement level was the highest of all categories, when patients participated in developing clinical guidelines with a median engagement score at level 6, partnership.

Looking at the research steps, the lowest level of engagement was found when patients were engaged in collecting data (step 4) with a median score of level 3, informing. In contrary, when patients did engage in analyzing and interpreting data (research step 5), although the number of studies was low, the median engagement level was much higher, at level 5, placation.

The studies found in this scoping review reveal an increase of patient engagement in earlier stages of the research process, for instance engaged in determining research topics. This way the research could be even more aligned to the needs and wishes of patients and thereby becoming more relevant. A well established way to engage patients and/or patient representative organizations is aiming to yield insights in the most important patients’ needs. A patient research agenda will give researchers, policy makers and patient representative organizations better insight in what problems patients experience, help clarify potential blind spots in the professional field and shape future research programs [38, 39]. However, this is not automatically followed by patient engagement at higher levels of participation in programming, collecting data, data analysis, publication of the results and implementation, especially of original research studies. For this reason, in the Netherlands for example, a Dutch agenda is being developed, in a collaboration between the Dutch Noonan Syndrome Foundation and national scientific and clinical professionals. Such an agenda will support the enhancement of both focus and relevance of future studies on Noonan syndrome spectrum disorders, especially when patients stay involved in the research process.

## Data Availability

All data generated or analysed during this study are included in this published article.
